# Complete Genome Sequence Analysis of *Nocardia brasiliensis* HUJEG-1 Reveals a Saprobic Lifestyle and the Genes Needed for Human Pathogenesis

**DOI:** 10.1371/journal.pone.0065425

**Published:** 2013-06-03

**Authors:** Lucio Vera-Cabrera, Rocio Ortiz-Lopez, Ramiro Elizondo-Gonzalez, Jorge Ocampo-Candiani

**Affiliations:** 1 Laboratorio Interdisciplinario de Investigación Dermatológica, Servicio de Dermatología, Hospital Universitario, U.A.N.L., Monterrey, N.L., México; 2 Universidad Autónoma de Nuevo León, Departmento de Bioquímica y Medicina Molecular, Monterrey, N.L., México; 3 Universidad Autónoma de Nuevo León, Centro de Investigación y Desarrollo en Ciencias de la Salud, Monterrey, N.L, México; University College Dublin, Ireland

## Abstract

*Nocardia brasiliensis* is an important etiologic agent of mycetoma. These bacteria live as a saprobe in soil or organic material and enter the tissue via minor trauma. Mycetoma is characterized by tumefaction and the production of fistula and abscesses, with no spontaneous cure. By using mass sequencing, we determined the complete genomic nucleotide sequence of the bacteria. According to our data, the genome is a circular chromosome 9,436,348-bp long with 68% G+C content that encodes 8,414 proteins. We observed orthologs for virulence factors, a higher number of genes involved in lipid biosynthesis and catabolism, and gene clusters for the synthesis of bioactive compounds, such as antibiotics, terpenes, and polyketides. An in silico analysis of the sequence supports the conclusion that the bacteria acquired diverse genes by horizontal transfer from other soil bacteria, even from eukaryotic organisms. The genome composition reflects the evolution of bacteria via the acquisition of a large amount of DNA, which allows it to survive in new ecological niches, including humans.

## Introduction

Actinobacteria are gram-positive organisms that are ecologically important in nature as re-cyclers of organic matter, including cellulose from plants and chitin from insects. Many actinobacteria are branched and may produce exospores. Actinobacteria are important in medicine because they produce many biological active compounds. Since Waksman described actinomycin in 1940, many antibiotic, cytostatic and immunosuppressive compounds have been obtained from these organisms [1]. One of the subgroups, *Corynebacterineae,* is characterized by the production of mycolic acids that provide strength to the bacterial cell wall. Included in this sub-order are the families *Corynebacteriaceae*, *Dietziaceae*, *Gordoniaceae*, *Mycobacteriaceae*, *Nocardiaceae*, *Tsukamurellaceae*, and *Williamsiaceae,* which include specialized human pathogens, such as *Mycobacterium tuberculosis*, *M. leprae*, and *Corynebacterium diphtheriae* [2]. The *Nocardiaceae* family includes the genera *Nocardia* and *Rhodococcus*. The latter is an animal pathogen, particularly found in horses and immunodepressed human patients [3], [4].

**Figure 1:**
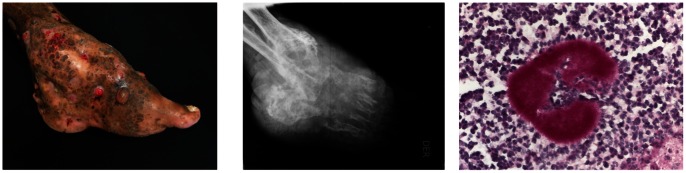
Mycetoma of the foot from *Nocardia brasiliensis* showing the characteristic triad of tumefaction, fistulae and microcolonies. Central image, an X-ray analysis of the ankle and foot region showing the severe destruction of bones. Right image, a microcolony of *N. brasiliensis* stained with PAS surrounded by a PMN infiltrate.

Nocardia species produce pulmonary, cutaneous and subcutaneous human diseases [5]. The most commonly isolated species include *N. brasiliensis, N. farcinica*, *N. cyriacigeorgica*, and *N. nova* [6], [7], [8]. Pulmonary nocardiosis has been reported particularly in patients with debilitating underlying conditions, such as organ transplant, leukemia, and diabetes. Mycetoma is a subcutaneous infection with differential histological and clinical characteristics [9]. There is an increase of the volume of the region affected, generally the limbs, and may affect muscle and fascia; in old and extended lesions bone destruction can be produced [Fig. 1]. The subcutaneous infection drains through the skin via fistulae discharging a serous purulent liquid. Generally, mycetoma occurs though a minor trauma with thorns, exposure of cutaneous lesions to soil, implantation of wood in the back, or even car accidents [9], [10]. Histologically, microcolonies of the agent, composed of a tightly branched mass of filaments, are observed in microabscesses surrounded by fibrous tissue. Patients do not report pain and are generally immunocompetent. Actinomycetes producing mycetoma include species of *Nocardia*, *Actinomadura*, *Streptomyces* and *Nocardiopsis*. Because they are soil bacteria, the etiologic species distribution depends on the geographical region. *A. madurae* and *A. pelletieri* are more commonly reported in Africa and India [9]. In America, *Nocardia*, particularly *Nocarda brasiliensis*, is the most abundant etiologic agent. In Mexico, *N. brasiliensis* is responsible for approximately 86% of cases.

The immunological mechanisms involved in actinomycetoma, as well as the virulence factors of *Nocardia*, are poorly understood, primarily because of a lack of tools to study them. Molecular genetic techniques have proved to be an excellent means of studying phylogenetic relationships, as well as the biological and pathogenic properties of bacteria, for both human pathogens and industrial organisms [11]. To elucidate the virulence mechanisms and biological properties of *N. brasiliensis,* we previously determined the complete genome sequence of *N. brasiliensis* HUJEG-1 (ATCC 700358), a strain used by our group in many immunological and antimicrobial assays [12], [13], [14]. The WGS was annotated in GenBank under the number NZ_AIHV00000000.1 and comprises 53 contigs [15]. Now, we have prepared the complete physical map by obtaining an optical map (OpGen Inc., Gaithersburg, Maryland) using pulse field electrophoresis, fixation and digestion with *Bgl*II and labeling of cut fragments with several fluorescence tags in order to obtain a restriction map. The contigs were aligned using this restriction map using MapSolver™ software and deposited in GenBank under the reference number NC_018681.1. Herein, we present an *in silico* analysis comparing the *N. brasiliensis* HUJEG-1 genome sequence with other available actinobacteria genomes, including other *Nocardia spp*.

## Results

### General Characteristics of the *N. brasiliensis* ATCC700358 Genome

**Figure 2:**
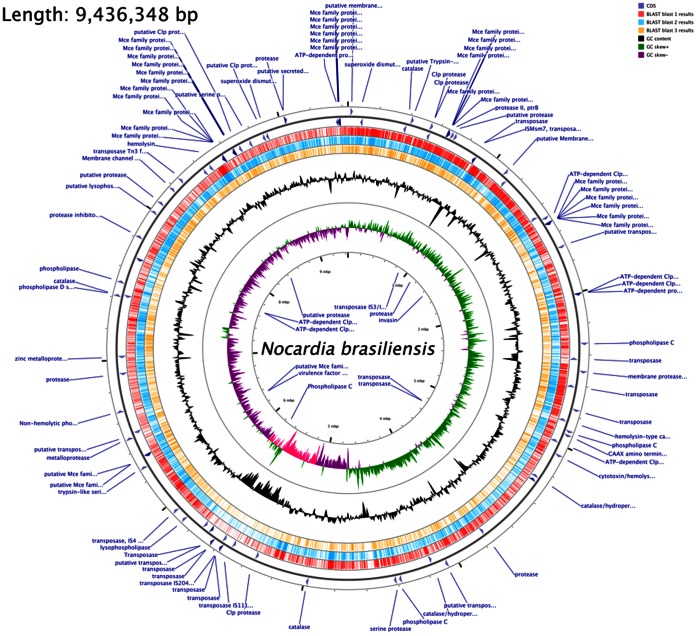
Physical map of the *N. brasiliensis* chromosome and gene clusters for putative virulence genes. The outer scale is numbered in megabases starting from the *dnaA* gene. The outermost two circles represent the position and strand direction of putative virulence genes and transposases. Circles 3, 4, and 5 represent a BLAST comparison with the complete genome of *Nocardia farcinica* IFM 10152 (NC_6361.1, red), *Rhodococcus equi* 103S (NC_014659.1, cyan) and *Mycobacterium tuberculosis* H37Rv (NC_000962.2, yellow), respectively. Circle 6, GC content; circle 7, GC bias. In magenta, we show the fragment with different G+C% (between nucleotide 5,126,000 and nucleotide 5,800,000).

In Figure 2, we show the physical map of the complete genome. Only one large contig (NZ_AIHV000025; 59,711-bp) could not be found in the optical map, and therefore, it is believed to be of extra-chromosomal origin. The total genome size is 9,436,348-bp, with a G+C% of 68. The genome encodes 51 tRNA, three copies of the 16S-23S-5S rRNA operon, and 8,414 predicted protein-coding sequences. Hypothetical proteins were predominant (5,745/8,414 proteins). In addition, 2,888 of the ORFs could be annotated using the BLAST program. Interestingly, there is a zone of about 600,000-bp starting at about nucleotide 5,126,00 to nucleotide 5,800,000 with a lower G+C % (63–65%). When analyzing this DNA stretch by using the internet program BLAST, very little homology with any gene in the GenBank library was observed, and we observed less than 10 genes that were similar in two other complete *Nocardia* genomes, *N*. *farcinica* IFM102 and *N. cyriacigerogica* GUH-2. Recently, the complete WGS of 26 *Nocardia* species, including one *N. brasiliensis* isolate, were released to GenBank. Surprisingly, this fragment was not observed in any of these genome sequences. It is possible that this fragment was acquired by *N. brasiliensis* HUJEG-1 by horizontal transfer, which has been observed in soil bacteria [16], although a transferred fragment this large has not been reported.

At the time of the release of the *N. brasiliensis* HUJEG-1 WGS sequence, only two *Nocardia* genomes had been published, *N. farcinica* IFM 10142 and *N. cyriacigeorgica* GUH-2, both belonging to the previously named *N. asteroides* complex [17], [18]. The reported sizes were approximately 6 MB (Table 1). Thus, we were surprised to find a 3.4-Mb larger genome. When comparing the complete genomes of pathogenic versus non-pathogenic bacteria, it has been observed that organisms that are more adapted to humans tend to have a smaller genome size because the bacteria eliminate those genes that are not needed for their parasitic lifestyle, such as the reduction in size of the genomes of the human pathogen *Mycobacterium leprae* and the filarial symbiont *Wolbachia spp*. [19], [20]. In our case, we found the opposite: a human pathogen with a large genome. When we compared the genome size among *Nocardia spp*., we observed sizes from 6.96 Mb for *N. asteroides* NBRC 15531 to 10.45 Mb for *N. jiangxiensis* NBRC 101359. *N. brasiliensis* NBRC 14402 (ATCC 19296) has a genome size of 8.9 Mb, which is similar to that of our strain. It appears that *N. brasiliensis* is not yet a specialized human pathogen and, instead, is a soil bacteria that occasionally affects humans.

**Table 1:** Comparison of genomic features of *Nocardia brasiliensis* and other bacteria.

	GenBank number	Size (Mb)	GC%	CDS	rRNA	tRNA	Genes
*Mycobacterium leprae* TN	NC_002677.1	3.27	57.8	1,605	3	45	2,770
*Mycobacterium tuberculosis* H37Rv	NC_000962.2	4.41	65.6	4,003	3	45	4,062
*Mycobacterium abscessus* ATCC 19977	NC_010397.1	5.07	64.1	4,920	3	47	4,970
*Mycobacterium smegmatis* str. MC2 155	NC_008596.1	6.99	67.4	6,717	6	47	6,938
*Nocardia farcinica* IFM 10152	NC_006361.1	6.29	70.7	5,934	9	53	5,998
*Nocardia cyriacigeorgica* GUH-2	NC_016887.1	6.19	68.4	5,477	9	49	5,560
*Nocardia brasiliensis* HUJEG-1	NC_018681.1	9.44	68	8,414	6	51	8,471
*Rhodococcus equi* ATCC 33707	NZ_CM001149.1	5.26	68.7	5,030	15	52	5,105
*Streptomyces griseus subsp. griseus* NBRC 13350	NC_010572.1	8.55	72.2	7,136	18	66	7,224
*Micromonospora aurantiaca* ATCC 27029	NC_014391.1	7.03	72.8	6,222	9	52	6,361
*Amycolatopsis mediterranei* U32	NC_014318.1	10.24	71.3	9,228	12	52	9,292
*Escherichia coli* O157:H7 str. EC4115	NC_011353.1	5.57	50.5	5,315	22	110	5,891

**Figure 3:**
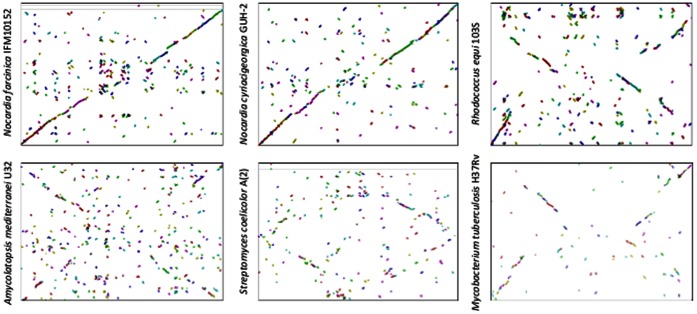
Syntenic dot plot of *N. brasiliensis* ATCC 700358 genome against *Nocardia farcinica* IFM10152 (a), *Nocardia cyriacigeorgica* GUH-2 (b), *Rhodococcus equi* 103S, *Amycolatopsis mediterranei* U32, *Streptomyces coelicolor* A(2), and *Mycobacterium tuberculosis* H37Rv genomes. Dots represent a reciprocal best match by BLASTP comparison. The *x*-axis corresponds to the *N. brasiliensis* genome plotted against the rest of the genomes (*y*-axis). Inclination to the right corresponds to ORFs in same direction. An inclination to the left corresponds to an opposite direction. The highest homology in the *Nocardia* species was found at about the *dnaA* site. In each case, genome coverage was 30, 30, 11, 7, 5 and 4%.

To attain a macro view of the genetic relationships with other bacteria, we compared the *N. brasiliensis* genome sequence with those of other actinomycetes, including *N. farcinica* 10152, *N. cyriacigeorgica* GUH-2, *Rhodococcus equi* 103S, *Amycolatopsis mediterranei* U32 (former *Nocardia mediterranei*, a rifampicin producer), *Streptomyces coelicolor* A(2) (antibiotic producer) and *Mycobacterium tuberculosis*, a recognized human pathogen. In Figure 3, we show a syntenic dot plot of the genomes of these species. A close relationship was observed with *N. cyriacigeorgica* and *N. farcinica*, with a higher density particularly at the end and the beginning of the chromosome (DNA core). As expected, there was more homology with another *Nocardiaceae* (*Rhodococcus*) than with the other bacteria, although a higher homology of the *A. mediterranei* and *S. coelicolor* genomes was observed than with another *Corynebacterineae*, such as *M. tuberculosis*. This finding may be explained by the fact that *M. tuberculosis* has evolved to be an almost exclusively human pathogen and has lost many of its soil inhabitant characteristics.

### Putative Virulence Factors

There have been several experimentally described virulence factors of *Nocardia,* including catalase, superoxide dismutase, cell-wall lipids, and proteases, as well as some immunodominant antigens, using mainly *N. cyriacigerogica* GUH-2 (formerly *N. asteroides*) and *N. brasiliensis* HUJEG-1 [21], [22], [23], [24], [25].

Catalases have been proved, in *N. cyriacigeorgica* and in other microorganisms, to be important in detoxifying the H_2_0_2_ produced by phagocytes. In *N. brasiliensis*, a catalase was described as the target of the humoral response in patients suffering mycetoma. At that time, the catalase was named P61 or *katN* [24]. *katN* is encoded by O3I_001640, and the closest ortholog proteins are found in *N. cyriacigeorgica* GUH-2 (87%) and in *M. abscessus* (85%). *N. farcinica* IFM 10152 has a lower-homology ortholog, nfa27070 (*katE*, 41%). Four more catalases were observed (Table 2), one of them very similar to *katG* of *M. tuberculosis* (77% identity, O3I_014530). The catalase gene O3I_032795 appears to be the most specific for *N. brasiliensis*, with a protein homology of 71% with *N. transvalensis* and of <30% with *N. farcinica* and *N. cyriacigeorgica* (Table S1).

**Table 2:** Distribution of putative virulence factors among actinomycetes with complete genome sequence.

	Catalase	Superoxide dismutase	Phospholipase C	Hemolysin	Protease	Chitinase
*Mycobacterium leprae* TN	0	2	0	0	0	0
*Mycobacterium tuberculosis* H37Rv	1	2	4	0	0	1
*Mycobacterium abscessus* ATCC 19977	4	3	2	1	23	0
*Mycobacterium smegmatis* str. MC2 155	5	1	0	0	0	0
*Nocardia farcinica* IFM 10152	4	2	0	2	29	0
*Nocardia cyriacigeorgica* GUH-2	3	2	0	2	35	0
*Nocardia brasiliensis* HUJEG-1	5	2	5	4	32	3
*Rhodococcus equi* ATCC 33707	4	4	4	4	25	4
*Streptomyces griseus subsp. griseus* NBRC 13350	4	2	5	0	52	10
*Micromonospora aurantiaca* ATCC 27029	2	4	3	2	10	1
*Amycolatopsis mediterranei* U32	2	1	7	3	64	13
*Escherichia coli* O157:H7 str. EC4115	3	3	0	0	0	0

As an external control we used *Escherichia coli*.

Superoxide dismutases are enzymes that are important for destroying deleterious superoxide and singlet O^−^_2_ ions that are produced during intracellular killing by phagocytes. This function has been demonstrated using *N. cyriacigeorgica* GUH-2 [22]. *N. brasiliensis* possesses two SODs: O3I_000385, which is very similar to *N. farcinica* and *N. cyriacigeorgica* GUH-2 *sodA* (97%), and other nocardial and mycobacterial species. The SOD gene O3I_039690 encodes for a protein that is similar only to *N. farcinica* and *N. cyriacigeorgica* GUH-1 (74 and 78% homology, respectively).

Phospholipase C proteins can be important virulence factors in tissue-destroying organisms such as *N. brasiliensis*, as has been demonstrated for other microorganisms [26], [27], [28]. We observed four phospholipase C proteins in the genome. O3I_010265 is quite specific for this microorganism. The closest protein (54% similar) is found in *Amycolatopsis mediterranei*. When compared to *Nocardia spp*, only the genome of *N. tenerifensis* contains a similar orthologous protein (87%). The other *Nocardia spp* genomes contained proteins with less than 51% homology. The phospholipase C gene O3I_012930 is very similar to the orthologs found in *Nocardia spp* (up to 87%), as well as in some *Gordonia* and *Rhodococcus* species. Phospholipase C O3I_019520 and O3I_025065 are even more specific to *N. brasiliensis*, with low observed homology to orthologs in other Nocardia species (<37%, except *N. transvalensis at* 62%) and other *Corynebacterineae*. No proteins with a significant E value were observed in the genomes of *N. farcinica* IFM10152 or *N. cyriacigeorgica* GUH-2. It appears that these phospholipases are specific to *N. brasiliensis* and that they may play an important role in *N. brasiliensis* pathogenesis.

Hemolysins are toxic proteins important in bacterial pathogenesis [29], [30]. The *N. brasiliensis* HUJEG-1 genome encodes 4 hemolysins. O3I_012605 is exclusive to *N. brasiliensis*. The gene is not present in *N. brasiliensis* NBRC 14402 or in the rest of the *Nocardia spp*, except *N. tenerifensis* where a similar protein (72%) is observed. The hemolysin O3I_037730 is present in *N. brasiliensis* NBRC 14402, with similar proteins in *N. tenerifensis* (86%), and low-homology proteins are present in the rest of *Nocardia spp* and other soil bacteria (<45%). The other two hemolysins have similar proteins in *Nocardia spp* and other actinobacteria. Mycetoma cases differ in extent and dissemination; these differences could be explained by the presence of more destructive enzymes in some strains, obtained from other innocuous soil bacteria by horizontal transfer.

Invasin is a protein that is used by several microorganisms (including *Yersinia pestis* and *Y. entreocolitica*, *Helycobacter jejuni*, *Plasmodium spp*) [30], [31], [32] to attach and penetrate into host cells. *Nocardia* is an intracellular facultative microorganism and possesses an invasin gene, O3I_027570, with a similar protein in *N. farcinica* (72%) and *N. cyriacigeorgica* GUH-2 (73%). Because all Nocardia are intracellular facultative cells, they most likely use this protein to attach to cells.

*N. brasiliensis* is identified by conventional methods from other *Nocardia spp* by analyzing the differential hydrolysis of compounds, including tyrosine, hypoxanthine, adenine and casein [33]. Although the profiles may vary, *N. brasiliensis* is the only casein-positive species. Surprisingly, we found not one but 32 proteases and one protease inhibitor. Most of the proteases have orthologs in other nocardial or other actinomycetes species. Proteases encoded by O3I_030410 and O3I_002340 presented homologies of 85–96% in many Nocardia species orthologs. These genes may constitute highly conserved genes, particularly the latter, with a homology of 93% to the phylogenetically distant *M. leprae*. In contrast, the protease gene O3I_013280 is quite specific, particularly after amino acid 111, where the homology with other Nocardia species is close to zero. The closest proteins are from *N. brasiliensis* NBRC 14402 (94% identity) and *N. tenerifensis* (57% identity). When compared by BLAST with other bacteria, a similar finding was observed, with the highest similarity shown to *Bifidobacterium angulatum* (49%). It is possible that O3I_013280 (228-amino acids long) was generated by homologous recombination from the huge O3I_002340 (772-amino acids long), given their homology for the first 111 amino acids (48%). Although not yet proved, given its specificity, it is possible that O3I_002340 codes for the caseinase utilized to differentiate *N. brasiliensis* from other *Nocardia spp*.

Free-living bacteria need to process many materials, including oligo elements, metals, and nutrients. In addition, they also need to release cell-wall synthetic materials, toxic compounds or other components. ABC transporters are specialized proteins that perform these functions [34]. The *N. brasiliensis* HUJEG-1 genome encodes 516 of these proteins compared to 150 encoded by *N. cyriacigeorgica* GUH-2; 139, *N. farcinica* IFM 102; 217, *Streptomyces coelicolor*; 458, *Amycolatopsis mediterranei* U32; and <100, *Mycobacterium tuberculosis*. In this regard, *N. brasiliensis* more resembles a soil bacteria than a pathogenic bacteria.

Mammalian cell entry proteins (mce) are essential for *M. tuberculosis* virulence. Their importance was first demonstrated by transferring the *Mce1* gene of *M. tuberculosis* to *Escherichia coli,* which produced an *E. coli* strain with the ability to attach and enter phagocytes, features not previously possessed [35], [36]. *N. brasiliensis* HUJEG-1 possesses 33 genes encoding mce proteins distributed in six operons, with four of them grouped in arrays of six ORFs (Table 3). All of these are also present in *N. brasiliensis* NBRC 14402 and have ortholog genes in many other *Nocardia* species and other *Corynebacterineae,* including *Mycobacterium*, *Rhodococcus*, *Gordonia*, and *Tsukamurella*. They have been demonstrated to be important in the virulence of *Mycobacterium tuberculosis*, as well as for transmembrane transportation [37].

**Table 3:** Distribution of important genome features among actinomycetes with complete genome sequence.

	Mce proteins	PE/PPE/PGRS	Cytochrome P450	Protocatechuate dioxygenase	Homogentisate dioxygenase
*Mycobacterium leprae* TN	5	4	0	0	0
*Mycobacterium tuberculosis* H37Rv	21	176	20	0	0
*Mycobacterium abscessus* ATCC 19977	44	12	25	0	1
*Mycobacterium smegmatis* str. MC2 155	1	0	0	0	0
*Nocardia farcinica* IFM 10152	36	0	26	0	0
*Nocardia cyriacigeorgica* GUH-2	40	4	6	1	1
*Nocardia brasiliensis* HUJEG-1	33	3	58	2	1
*Rhodococcus equi* ATCC 33707	13	4	4	3	2
*Streptomyces griseus subsp. griseus* NBRC 13350	6	0	26	0	1
*Micromonospora aurantiaca* ATCC 27029	0	0	14	0	1
*Amycolatopsis mediterranei* U32	0	0	54	3	0
*Escherichia coli* O157:H7 str. EC4115	0	0	0	0	0

As an external control we used *Escherichia coli*.

PE (proline-glutamate) and PPE (proline-proline-glutamate) family proteins constitute approximately 10% of the *M. tuberculosis* genome [38]. These proteins possibly provide for a high level of antigenic variability. In the *N. brasiliensis* HUJEG-1 genome, we observed only three PPE genes, O3I_000480, O3I_023795 and O3I_023865, which were 394, 391 and 488 amino acids in length, respectively. The first PPE protein is identical in *N*. *brasiliensis* NBRC 14402 and has orthologs in other Nocardia species, including *N. farcinica* IFM 10152 and *N. cyriacigeorgica* GUH-2 (66 and 62% identity, respectively). Other bacteria having orthologs for this protein include *Rhodococcus* and *Mycobacterium spp*. Interestingly, in this protein, after amino acid 228, homology significantly decreases. In contrast, PE_PPE O3I_023795 is almost exclusively found in *N. brasiliensis* HUJEG-1 and in the first 251 amino acids has stretches of low homology to *N. cyriacigeorgica* and *Mycobacterium spp*. The rest of the sequence has no homology to any other protein according to a BLAST analysis. The sequence is not present in *N. brasiliensis* NBRC 14402. PPE O3I_023865 is also not present in *N. brasiliensis* NBRC 14402 and has zones of homology with proteins of several *Nocardia spp* only until amino acid 272 (up to 39% homology). The rest of the sequence presents only scarce homology. Only *N. pneumonia* and *N. abscessus* have homology throughout the entire sequence. The gene is exclusive to *Nocardia spp*, with no similar orthologs in any other bacteria according to a BLAST analysis. The division of these proteins into homology dominions indicates a possible recombination origin among them.

### DNA Duplication

Gyrases are type II topoisomerases that help helicases to unwind double-stranded DNA. *N. brasiliensis* HUJEG-1 possesses one *gyrA* (O3I_000030) and one *gyrB* (O3I_000025) subunit, plus one additional *gyrB* subunit (O3I_029325). As in most bacterial species, in *N. brasiliensis* HUJEG-1, the *gyrA* and *gyrB* genes are situated close to the replication origin. The second B subunit gene is located at nucleotide 6,674,508. The three proteins exist also in *N. brasiliensis* NBRC 14402; *gyrA* and *gyrB* are very similar in other *Nocardia spp* (88–95%) and *Mycobacterium* (84–86%), including *M. tuberculosis*. The second B subunit is less similar among *Nocardia* and mycobacterial *gyrB* genes (approximately 74%). The presence of three gyrase genes in *N. brasiliensis* instead of two may explain the natural resistance of the bacteria to quinolones, such as ciprofloxacin [39]. Third-generation, more potent drugs, such gatifloxacin and moxifloxacin, are active against *N. brasiliensis* in vitro. In to regard the number of gyrase genes, other soil-inhabiting bacteria such as *Amycolatopsis mediterranei* possess four gyrase genes.

### Secondary Metabolites

Non-ribosomal peptides are secondary metabolites with cyclic or branched structures, which are not synthesized by ribosomal mRNA but instead by non-ribosomal peptide synthetases. These proteins synthesize only one specific peptide, and they can include D-amino acids and catalyze chemical changes such as glycosylations and acylations. These peptides have a wide spectrum of biological activities, including functions as immunosuppressants, toxins, fluorescent pigments, and cytostatics, such as garamycin, vancomycin, and cyclosporin [40]. *N. brasiliensis* HUJEG-1 possesses approximately 30 genes encoding for non-ribosomal peptide synthetases, including some with very high calculated molecular weights, such as 03I_037910 (1,570,367 Da). The genes 03I_007380 and 03I_007385 code for the synthesis of a compound similar to pyoverdin, the typical green pigment of *Pseudomonas aeruginosa*. The produced product is similar to pyoverdin from *Frankia* (47%) and similar to peptides from bacteria belonging to the *Pseudomonadaceae* family, e.g., *Aeromonas*, *Burkholderia* and *Pseudomonas* (approximately 36% homology) [41].

Polyketides are secondary metabolites that are produced by the decarboxylative condensation of malonyl-CoA derived extender units. Polyketides possess a wide variety of biological properties as antibiotics, anti-cancer compounds, insecticides, and so forth [42]. Among these compounds are macrolides, such as anthramycin; polyene antifungals, such as amphotericin B; and toxic compounds, such as aflatoxins. *N. brasiliensis* HUJEG-1 encodes 20 related polyketide synthase genes. Some of these genes are very similar to proteins in *N. cyriacigeorgica* GUH-2 and *N. farcinica* IFM10152. In contrast, the polyketide synthase gene O3I_007465 presents very low homology to nfa17160 of *N. farcinica* IF 10152 and *N. cyriacigeorgica* GUH-2 *pknG* (approximately 18% coverage and 36% identity in both cases). It also encodes a polyketide synthase that is highly homologous (80%) to a protein from *Streptomyces venezuelae* involved in the synthesis of jadomycin, a polyketide antibiotic. Nocardia have been the source of many bioactive substances [43], and it will be important to determine the role of these enzymes in the synthesis of *N. brasiliensis* polyketide compounds.

Terpenes are derived biosynthetically from units of isoprene and were originally isolated from the resin of the turpentine tree [44]. Terpene compounds in *Nocardia* have been found to have antibiotic and cytostatic properties [44], [45]. *N. brasiliensis* HUJEG-1 encodes for 4 terpene synthases with no orthologs in *N. cyriacigeorgica* GUH-2 or *N. farcinica* IFM10152 but with orthologs in other actinobacteria genera, such as *Saccharopolyspora*, *Streptomyces* and *Amycolatopsis*.

The *N. brasiliensis* HUJEG-1 genome encodes nine genes that are involved in antibiotic synthesis, most of them with orthologs in *N. cyriacigeorgica* GUH-2 and *N. farcinica* IFM 10152. The antibiotic synthesis genes O3I_005645, O3I_014615, and O3I_014620 are not found in *N. cyriacigeorgica* GUH-2 or *N. farcinica* IFM 10152. In contrast, they have orthologs in *Bacillus*, *Paenibacillus* or *Brevibacillus*. Other antibiotic biosynthesis genes that are present in the *N. brasiliensis* HUJEG-1 genome include those involved in the synthesis of erythromycin, hygromycin, puromycin, saframycin, streptomycin and tetracenomycin.

When analyzing the genomes of *N. brasiliensis* HUJEG-1, *N. farcinica* and *N. cyriacigeorgica* using the Antibiotics and Secondary Metabolites Analysis Shell (antismash) software [http://antismash.secondarymetabolites.org], which searches genomes looking for secondary metabolite gene clusters, we found 47 clusters in *N. brasiliensis* HUJEG-1, 16 in *N. farcinica* IFM 10152 and 21 in *N. cyriacigeorgica* GUH-2. *N. brasiliensis* uses approximately a quarter of its genome to synthesize secondary metabolites (2,157,079-bp). In contrast, *N. farcinica* IFM 10152 uses 833,872 bp and *N. cyriacigeorgica* GUH-2 uses 985,767 bp of their respective genomes to encode for these metabolites. *M. tuberculosis* H37Rv uses 778,422 bp. When analyzing the cluster locations in the genome, half of them are situated between nucleotide 3,000,000 and nucleotide 5,800,000 (Fig. S1). In this zone, only 1 out of 21 clusters have orthologs found in *N. farcinica* or *N. cyriacigeorgica*. In contrast, in the clusters found in the “DNA core” zone of the genome (approximately three megabases before and after the *dnaA* gene), half of the genes have orthologs in either *N. farcinica* or *N. cyriacigeorgica*, or in both (13 out of 26).

### Oxidative Pathways

Cytochromes P450 (CYPs) are important proteins that catalyze the oxidation of many substrates. CYPs are hemoproteins, enzymes containing a heme prosthetic group, and thus these proteins have a characteristic red color. *N. brasiliensis* HUJEG-1 possesses abundant CYPs (57 genes), and some of them have many orthologs, such as O3I_016325, which has similar proteins in *N. cyriacigeorgica* GUH-2 and *N. farcinica* IFM 10152, with homology of approximately 68 and 78% respectively. This CYP has also many homologs distributed in *Streptomyces spp.*, such as *S. coelicolor* (61%). Other CYPs are more specific to *N. brasiliensis,* with a homology of less than 30% with *N. cyriacigeorgica* (coverage 37%) and with a high similarity to CYPs in other actinobacteria, such as *A. mediterranei* (91% homology, 57% coverage). The high abundance of CYP reflects its large metabolic capacity, and homology with other soil bacterial proteins indicates its possible acquisition via horizontal transfer. This abundance of CYPs also may explain the natural resistance of *N. brasiliensis* to most azolic compounds, which mainly target the cytochrome P450 enzymes homologues to 14-alfa-sterol demethylases [46], [47]. For instance, *Candida albicans* possesses only two cytochrome P450 proteins and is highly susceptible to azoles.

We also observed genes encoding antioxidants, such as thioredoxins (seven genes), and low-molecular weight non-heme iron proteins, such as rubredoxin and rubrerythrin, in the genome of *N. brasiliensis*.

### DNA Elements

Transposases are enzymes that catalyze the movement of a transposon from one location in a chromosome to another via a copy and paste system [48]. Transposases can provide important plasticity and variability to bacterial genomes [49]. The *Nocardia brasiliensis* HUJEG-1 genome contains 21 genes encoding transposases. Some of them, such as O3I_014660, are shared with *N. cyriacigeorgica* GUH-2 (82%) and *N. farcinica* IFM 10152 (85%) and are similar to proteins found in other actinomycetes, such as IS*994* of *Renibacterium salmoninarum* (68%) and IS*6110* of *Mycobacterium tuberculosis* (75%). In contrast, O3I_024350, a transposase of the IS204/IS1001/IS1096/IS1165 family, is not present in either *N. cyriacigeorgica* GUH-2 or *N. farcinica* IFM 10152 but is similar to proteins in *Streptomyces violaceusniger* Tu 4113 (51%), which supports an external origin for these insertional elements. Insertion sequences have been used widely to subtype bacterial species, but unfortunately, in this strain, we only observed single copies of these elements, thus eliminating a possible use for subtyping, unless other *N. brasiliensis* strains have a variable number, as has been observed for IS*6110* of *M. tuberculosis,* where the number of copies can range from 0 to 25 [48]. Interestingly, eight of the transposases are present in a hot spot (Fig. 2) that is located in a stretch (mentioned above) from nucleotide 5,126,00 to nucleotide 5,800,000, in the fragment with different G+C content, but are not present in *N. brasiliensis* ATCC 19296. These transposases may have been acquired by horizontal transference. The abundance of transposase may partially explain the acquisition of this fragment.

During phage infection, some of the virus enters a lysogenic cycle, and the phage DNA is integrated into the bacterial chromosome [50]. In the *N. brasiliensis* HUJEG-1 genome, at least six genes associated with phages are present. In comparison with *M. tuberculosis* (two prophages) or *N. farcinica* (three prophages), where these genes are found in clusters, including the ORFs for each viral function, in *N. brasiliensis*, these genes are dispersed in the genome, and only two sequences encoding phage integrases are adjacently located. This may be explained by the elimination of the remaining phage genes or the fact that the genes have very low homology to other sequences reported in GenBank.

In response to external DNA invasion, either by plasmids or phages, bacteria have developed a protective system based on the recognition of foreign DNA using Clustered Regularly Interspaced Short Palindromic Repeat (CRISPR) sequences together with CRISPR-associated proteins (CAS) [51]. *N. brasiliensis* HUJEG-1 possesses one gene encoding for a Cas5E protein (O3I_023695) with orthologs in *N. farcinica* IFM 10152, *N. cyriacigeorgica* GUH-2 and other soil bacteria.

### Antimicrobial Resistance

*N. brasiliensis* actinomycetoma is difficult to treat, in part because of the natural resistance of this microorganism to many drugs. As described above, *N. brasiliensis* HUJEG-1 possesses many ABC transporters that may facilitate the elimination of toxic compounds, including drugs and metabolite derivatives. In addition, *N. brasiliensis* is resistant to most beta-lactams, even after adding strong anti-beta lactam inhibitors, such as tazobactam or clavulanic acid [52], [53]. This resistance may be explained by the large amount of beta-lactamases that are encoded in the *N. brasiliensis* genome (n = 29). In contrast, *N. cyriacigeorgica* has 12 such genes, and *N. farcinica* has only one. Some *N. brasiliensis* beta-lactamases, such as O3I_003795, are highly conserved among *Corynebacterineae*, with 76% homology to *N. cyriacigeorgica* GUH-2 and *Rhodococcus* (77%) orthologs. Some others genes, such as O3I_003205, display a lower similarity to *N. farcinica* (35%) than to *Streptosporangium roseum* (69%) orthologs, suggesting that some of these beta-lactamase genes were acquired by horizontal transfer from other soil bacteria. The presence of extra genes that are the target of antimicrobials may be the basis for the resistance of *N. brasiliensis*. For instance, the presence of a second gyrase B and an extra copy of *rpoB* can be associated with antimicrobial resistance to quinolones and rifampin.

### Metabolism

Environmental microorganisms may use simple organic compounds such as alkanes or even substrates containing aromatic rings as nutrients [54], [55]. These compounds may be degraded using the protocatechuate and/or the homogentisate pathways, producing succinate-acetyl-CoA and fumarate-acetoacetate, which enter the general metabolism pathways for use in the catabolism or synthesis of compounds. *N. brasiliensis* can use both systems because the bacteria possess genes that encode both enzymes: protocatechuate 3,4-dioxygenase, in the alpha and beta subunits (O3I_021760 and O3I_021765), and a homogentisate 2,3-dioxygenase (O3I_039745). The protocatechuate 3,4-dioxygenase is very similar to other proteins from *Geodermatophilus* or *Saccharomonospora* (up to 74%). In contrast, they are not observed or have orthologs with very low homology to some of these genes in *N. cyriacigeorgica* GUH-2 and *N. farcinica* IFM 10152. The homogentisate 1,2 dioxygenase of *N. brasiliensis* is quite similar to an ortholog in *N. cyriacigeorgica* GUH-2 and *N. farcinica* IFM 10152 (89 and 83% homology). This enzyme is also conserved in other *Nocardiaceae*.

### Degrading Enzymes Production

Chitinases are enzymes that degrade glycosidic bonds in chitin and are a very useful enzyme for soil bacteria and fungi for degrading dead insects [56]. *N. brasiliensis* HUJEG-1 possesses three genes encoding chitinases, with no significant orthologs in *N. cyriacigeorgica* GUH-2 or *N. farcinica* IFM152. The chitinase genes O3I_019530 and O3I_029865 have very similar orthologs in *Streptomyces* and *Kitasatospora* (up to 80% homology). Interestingly, a third gene, O3I_036765, the closest homologous protein according to the BLAST analysis (37%), is from *Nasonia vitripennis,* a small wasp. *N. brasiliensis* has not been isolated from insects, and some insects may use antibiotics produced by symbiotic actinomycetes to keep their eggs free of bacterial infection [57]. There is only one report of the production of mycetoma after the sting of a yellow wasp. It would be interesting to study insects as a putative ecological niche or substrate for *N. brasiliensis* [58].

Plants leaves are protected by an external polyester layer that is composed of hydroxy and hydroxyl epoxy fatty acids named cutin [59]. Fungi and other plant eater organisms possess cutinases, enzymes that can degrade this polymer. *N. brasiliensis* HUJEG-1 has three genes, O3I_014370, O3I_015280 and O3I_017645, that encode cutinases. Orthologs are found in *N. cyriacigeorgica* GUH-2 and *N. farcinica* IFM 10152. The gene O3I_017645 is 43% homologous to the cutinase of *Phytophthora sojae*, an oomycete producing plant infestations, such as the famous potato epidemic in Ireland, which resulted in massive emigration to the U.S. *circa* 1845. *Mycobacterium tuberculosis* possesses a cutinase, similar to O3I_014370 (27 coverage, 34% homology), probably as a result of its history of being a soil living bacteria.

### General Metabolism

It is apparent from general genome inspection that *N. brasiliensis* possesses the complete pathways for glycolysis, pentose phosphate processing, and the tricarboxylic acid and glyoxylate cycles. The presence of a high number of oxidoreductases (approximately 150), dehydrogenases (n = 335), and oxygenases (approximately 150, including CYPs) reflects the aerobic metabolism of *N. brasiliensis*. Using these proteins, ATP is produced by NADH, and the ubiquinone chain is present. Even if Nocardia are typically an aerobic organism, genes for nitrate reductase (two operons), fumarate reductase and nitrite reductase, which are important enzymes in anaerobic phosphorylation, are also present. These genes might support growth in low vascularized abscessed tissue with reduced redox potential.

A large chromosome, such as the genome of *N. brasiliensis*, that contains the genetic information necessary to adapt to different environments, such as soil and possible insect and human hosts, requires an extensive regulatory system. Approximately 17 sigma factors, 11 anti-sigma factors, and 21 RNA polymerase sigma factors are predicted to exist in the *N. brasiliensis* genome. In a similar manner to *M. tuberculosis,* which possesses 11 genes for two-component histidine kinase sensors, *N. brasiliensis* also has a small number of these genes (n = 7), compared to >30 present in *E. coli*. *N. brasiliensis* also has 78 two-component system genes. This environmental signal transduction system is complemented by the presence of 30 predicted serine/threonine protein kinases, which are important in regulating apoptosis and cell division through the phosphorylation of specific proteins.

LuxR regulators are a group of bacterial helix-turn-helix (HTH) transcription factors that are involved in the regulation of many bacterial quorum-sensing (QS) mechanisms [60]. *N. brasiliensis* has a LuxR system, including a putative two-component system response regulator of the LuxR family protein together with 23 transcriptional regulators. These LuxR systems conduct a census of their own bacterial population, which is important for soil-inhabiting bacteria, as a great variety of species co-exist in the soil.

### Lipid Metabolism

Organisms belonging to the *Corynebacterineae* are characterized by possessing large amounts of lipids in their cell wall, including, fatty acids, lipo-oligosaccharides, phenolthiocerols, mycolic acids, and lipoarabinomannans. This trait is a hallmark of this suborder. In the *N. brasiliensis* genome, there is an abundance of enzymes that are involved in lipid catabolic and anabolic pathways, including 15 acyl-CoA synthetases, 6 long chain acyl-CoA synthetases, 12 enoyl-CoA hydratase/isomerases, twelve acetyl-CoA acetyltransferases and FadA/FadB beta-oxidation complex proteins (O3I_003935 and O3I_001385 genes), which complete the beta-oxidation of the fatty acids.

*Nocardia*, as with all *Corynebacterineae*, has an abundant amount of mycolic acids, (beta-hydroxy-alpha branched fatty acids) which can account for up to 60% dry weight in some species. The biosynthesis of mycolic acids precursors in *Corynebacterineae* requires two systems: a unique polypeptide multifunctional enzyme denominated FAS I and a FAS-II system composed of several enzymes [61], [62]. A FAS-I homolog is encoded in *N. brasiliensis* by O3I_007715, a 3,125-amino acid polyfunctional protein that is highly conserved in *N. cyriacigeorgica* GUH-2 (86% ), *N. farcinica* IFM 102 (86% ) and *M. tuberculosis* (63%). The products of FAS I serve as substrates for the FAS II system. In *M. tuberculosis*, the FAS II genes are clustered in two transcriptional units: the *mtfabD-acpM-kasA-kasB-accD6* and the *mabA-inhA* clusters. Malonyl-CoA:ACP transacylase (O3I_025190 in *N. brasiliensis*) transforms malonyl-CoA into malonyl-ACP. Beta-ketoacyl-ACP synthase III (*fabH* in MTB) condenses the acetyl-CoA that is produced by FAS I with malonyl-ACP to elongate fatty acids. In *N. brasiliensis,* there are 4 of these enzymes. The O3I_039605 and O3I_040210 genes encode for proteins homologous to *fabH* (55 and 51% homology, respectively). In a second step of the elongation, the beta-hydroxyacyl-ACP intermediate is dehydrated to form trans-2-enoyl-ACP by the ketoacyl-ACP reductase (*mabA* in MTB). *N. brasiliensis* possesses four of these ketoacyl-ACP reductases. O3I_027550 encodes a protein similar to *mabA* in *M. tuberculosis* (67%). The other three proteins have no significant homology with *M. tuberculosis* proteins. O3I_027550 is very conserved in *N. farcinica* IFM 10152 and *N. cyriacigeorgica* GUH-2 (85 and 73%, respectively). The final elongation step is carried out by the *inhA* gene, an NADH-dependent enoyl-ACP reductase. The equivalent in *N. brasiliensis* is an enoyl-(acyl carrier protein) reductase (O3I_027545), which is very conserved in *N. cyriacigeorgica* GUH-2 and *N. farcinica* IFM 10152 (90 and 91%), with less homology to MTB (59%).

The modification of the fatty acids includes the introduction of *cis* double bonds. In *S*. *pneumoniae,* a combination of *fabZ* and *fabM* is used to introduce a double bond in the nascent acyl chain. *N. brasiliensis* possesses two genes, O3I_026205 and O3I_026230, that encode a beta-hydroxyacyl-(acyl-carrier-protein) dehydratase (FabZ) with low homology with proteins from *N. cyriacigeorgica*/*N. farcinica* (<30%), *Halanaerobium hydrogeniformans* (32%) and *Nitratireductor aquibiodomus* (37%). No *fabZ-* or *fabA*-like proteins exist in *M. tuberculosis*. Instead, *M. tuberculosis* has three potential aerobic desaturases encoded by *desA1*, *desA2* and *desA3*. *N. brasiliensis* has 15 desaturases, including a phytoene (40-carbon carotene synthesis intermediate) desaturase. O3I_016590 is a homolog of MTB *desA1*(55%).O3I_034520 is similar to *erg3*, an MTB desataurase (Rv1814) (47%). The genes O3I_007130, O3I_035030 and O3I_035040 encode proteins similar to a linoleoyl-CoA desaturase (Rv3229c) (about 60%) of MTB. The other genes have no similarity to MTB proteins. Most of these desaturase enzymes have highly similar orthologs in *N. farcinica* IFM 10152 and *N. cyriacigeorgica* GUH-2 (>75%). Desaturase genes O3I_022675 and O3I_029100 have no significant orthologs in *N. farcinica* IFM 10152 or *N. cyriacigeorgica* GUH-2; the highest homology found was with proteins of *Streptomyces bingchenggensis* (49%) and *Micromonospora sp.* ATCC 39149 (63%), strongly suggesting that these desaturase genes were acquired by horizontal transfer.

Mycolic acids differ among the *Corynebacterineae* not only in the length of the fatty acids but also in modifications such as the presence of oxygenated functions, cyclopropanes, or double bonds. *N. brasiliensis* encodes for 7 cyclopropane fatty acid synthases. O3I_001400 is present in some MTB strains, such as CDC1551 (68% protein identity), but is not present in H37Rv. O3I_008300 is an ortholog (58%) of *cmaA1* of MTB. O3I_029080 is an ortholog (33%) of *cmaA2*, and O3I_034505 is an ortholog of *ufaA1* (54%). The other genes have homology to genes in soil bacteria and other *Nocardiaceae*. O3I_008300, in particular, has many orthologs among the *Mycobacterium* species with a protein homology close to 60%. The loss of cyclopropane rings has been associated with a loss of virulence in MTB [63]. Interestingly, the *Nocardia* species that affect most immunocompromised patients, such as *N. farcinica* and *N. cyriacigeorgica*, have genomes with only one cyclopropane fatty synthase.

S-adenosylmethionine (SAM) is used as a methyl donor to a *cis*-ethylenic precursor to produce cyclopropanes and a methyl branch adjacent to a *trans* double bond or a *trans* cyclopropane. *N. brasiliensis* encodes for four S-adenosylmethionine-dependent methyltransferases, all of which have similar orthologs in *N. farcinica* IFM 10152 and *N. cyriacigeorgica* GUH-2. Two of these methyltransferases, O3I_000195 and O3I_006220, have orthologs in MTB (hypothetical proteins Rv0329c [34%] and Rv1300 [51%]).

In MTB, the introduction of keto- or methoxy- groups is mediated by the enzyme methoxy mycolic acid synthase Hma (MmaA4). In *N. brasiliensis,* the equivalent ortholog gene is O3I_008300, which encodes for a protein that is annotated as a cyclopropane-fatty-acyl-phospholipid synthase and that is also identified as an SAM-dependent methyl transferase, with a higher homology to MTB proteins (49%) and other *Mycobacterium* species than to *N. farcinica* IFM 10152 and *N. cyriacigeorgica* GUH-2 proteins (34% in both cases).

Condensation is the final step in mycolic acids biosynthesis. This process is carried out in *Corynebacterium* and *Mycobacterium* by a polyketide synthase, pks13, together with the activation of the meromycolic chain (acyl-AMP ligase) and that carboxylation of the alpha branch (by an acyl-CoA carboxylase). In *N. brasiliensis,* we found an orthologous gene, O3I_000755, similar to MTB pks13 (53%), with orthologs in *N. farcinica* IFM 10152 and *N. cyriacigeorgica* GUH-2 (78% in both cases). In MTB, fadD32 catalyzes the production of acyl-AMP using free fatty acid as a substrate. *N. brasiliensis* has a fadD32 ortholog, O3I_000750, which is 61 and 60% similar to *fadD32* of MTB and *M. leprae*, respectively. O3I_000750 is also highly conserved in *Nocardiaceae*, with 92% homology to orthologs in *N. farcinica* IFM 10152 and *N. cyriacigeorgica* GUH-2. In a similar manner to *fadD32,* which is located beside pk13 in MTB, O3I_000750 is adjacent to O3I_000755 (an ortholog of pk13), demonstrating a highly conserved similarity in these essential pathways in the *Corynebacterineae*. The activation of the alpha branch in the mycolic acids is carried out by an acyl-CoA carboxylase (accdD4) in *Corynebacterium* and *Mycobacterium spp*, which is the final step before condensation is carried out by pk13. The ortholog of *accD4* in *N. brasiliensis* is an acyl-CoA carboxylase that is encoded by O3I_000760, which is 71% homologous to accD4 of MTB and 94% homologous to those of *N. farcinica* IFM 10152 and *N. cyriacigeorgica* GUH-2. This gene is located immediately after the pks13 ortholog, suggesting that these three enzymes have common transcriptional regulators.

After condensation, the mycolic acids must be transported to their final location in the cell wall. In MTB, this step is carried out by mycolyl-transferases called fbp proteins or Antigen85 complex [64]. The *N. brasiliensis* genome encodes 10 mycolyl-transferases. Three of these genes, O3I_000710, O3I_000715 and O3I_000720, are arranged in tandem. *N. cyriacigeorgica* GUH-2 has 5 mycolyl transferases, and *N. farcinica* IFM 10152 has three of them. The *N. brasiliensis* O3I_000720, O3I_015965 and O3I_000980 genes are orthologs of *fbpC* of MTB (approximately 40% homology). No orthologs of the MTB genes *fbpA*, *fbpB* or *fbpD* were observed. One of the *N. brasiliensis* mycolyl-transferases, O3I_027065, has many orthologs in *Corynebacterineae* but only for the first 256 amino acids. The rest of the sequence is similar only to an ortholog in *Corynebacterium variabile* and *Corynebacterium ulcerans,* indicating that to *N. brasiliensis* acquired this sequence by horizontal transfer from *Corynebacterium spp*.

## Discussion

The in silico analysis of the *N. brasiliensis* HUJEG-1 genome demonstrates the transfer of genomic components among environmental bacteria, such as *Corynebacterium*, *Bacillus*, and *Streptomyces*, and even DNA from an insect origin, varying in size from very large fragments (approximately 600,000-bp) to small stretches of DNA. As a result, the bacteria are able to infect human hosts, but unlike the elimination of DNA observed in professional intracellular actinobacteria, such as *M. leprae*, *N. brasiliensis* has one of the largest bacterial genomes reported. Bacterial evolution seems to produce not only organisms with complex DNA to evolve to larger organisms, such as plants or animals, but also organisms with reduced DNA that are highly dependent on their hosts, such as parasites or symbionts.

The study of the genomic properties that *N. brasiliensis* shares with other mycetoma producing actinobacteria, such as *Actinomadura* and *Streptomyces,* will allow us to separate the part of the genome that is involved in environmental survival and those genes that allow these bacteria to infect human hosts.

## Materials and Methods

### Genome Sequencing and Assembly

*Nocardia brasiliensis* HUJEG-1 was deposited in the American Type Culture Collection Institute and designated as ATCC700358. The bacteria used for genome sequencing were isolated from a single colony-purified stock that was kept at −70°C, and the genomic DNA was extracted directly from the expanded culture. The genome sequence was determined using the Roche/454 GS (FLX Titanium) sequencing platform (8-kb library). A total of 786,647 reads were obtained, providing about 27-fold genome coverage. The Roche/454 GS reads were assembled using Newbler 2.5.3 software (Roche Diagnostics, Branford, CT). The unclosed draft genome of *N. brasiliensis* HUJEG-1 was composed of 53 contigs, for a total 10.8 Mbp, with 68% G+C content. The physical map was constructed in part by comparison with another clone of *N. brasiliensis* HUJEG-1 and a WGS available in GenBank, *N. brasiliensis* ATCC 19296. The final assembly was performed using pulse field electrophoresis followed by fluorescence labeling of the cut fragments. An optical map of the separated and labeled DNA fragments was prepared using *Bgl*II digestion (OpGen, Gaithersburg, Maryland). The contigs were aligned using this restriction map using the MapSolver™ software. The physical map image (Fig. 2) was prepared by Jason Grant from the Department of Agricultural, Food and Nutritional Science (AFNS) Edmonton, Alberta, Canada using the CG view program [65]. The final sequence was deposited in GenBank under the reference number NC_018681.1.

### Genome Annotation and Analysis

The genome annotation was conducted with the NCBI Prokaryotic Genomes Automatic Annotation Pipeline (PGAAP). According to the NCBI, the PGAAP combines HMM-based gene prediction methods with a sequence similarity-based approach that combines a comparison of the predicted gene products to the non-redundant protein database, Entrez Protein Clusters, the Conserved Domain Database, and the COGs (Clusters of Orthologous Groups) [66]. Gene predictions were performed using a combination of the GeneMark and Glimmer programs [67], [68]. A short step resolving conflicts of start sites was conducted at this point. Ribosomal RNAs were predicted by sequence similarity searching using BLAST against an RNA sequence database and/or using Infernal and Rfam models. Transfer RNAs were predicted using tRNAscan-SE [69]. To detect missing genes, a complete six-frame translation of the nucleotide sequence was performed, and predicted proteins (generated above) were masked. All predictions were then searched using BLAST against all proteins from complete microbial genomes. Annotation was based on comparison to protein clusters and on the BLAST results. The Conserved Domain Database and Cluster of Orthologous Group information was then added to the annotation.

## Supporting Information

Figure S1 Genomic location of the 47 gene clusters found in Nocardia brasiliensis HUJEG-1 using Antibiotics and Secondary Metabolites Analysis Shell (antismash) software [http://antismash.secondarymetabolites.org]. Besides the map we show a comparative analysis of cluster 1, which is highly conserved among the Nocardiaceae, located between nucleotides 1682661 – 1725128 nt, and cluster 38, which is quite specific of *N. brasiliensis* and is located between nucleotides 6591347–6636992
(PPTX)

Table S1 Presence of ortholog genes of putative virulence factors of *N. brasiliensis* in other microorganisms.
(DOCX)
