# Supplementary figures and images for: Complete Genome Sequence Analysis of Nocardia brasiliensis HUJEG-1 Reveals a Saprobic Lifestyle and the Genes Needed for Human Pathogenesis
Source: PLoS One. 2013 Jun 3;8(6):e65425. doi: 10.1371/journal.pone.0065425 (PMC3670865; doi:10.1371/journal.pone.0065425)

## Slide 1
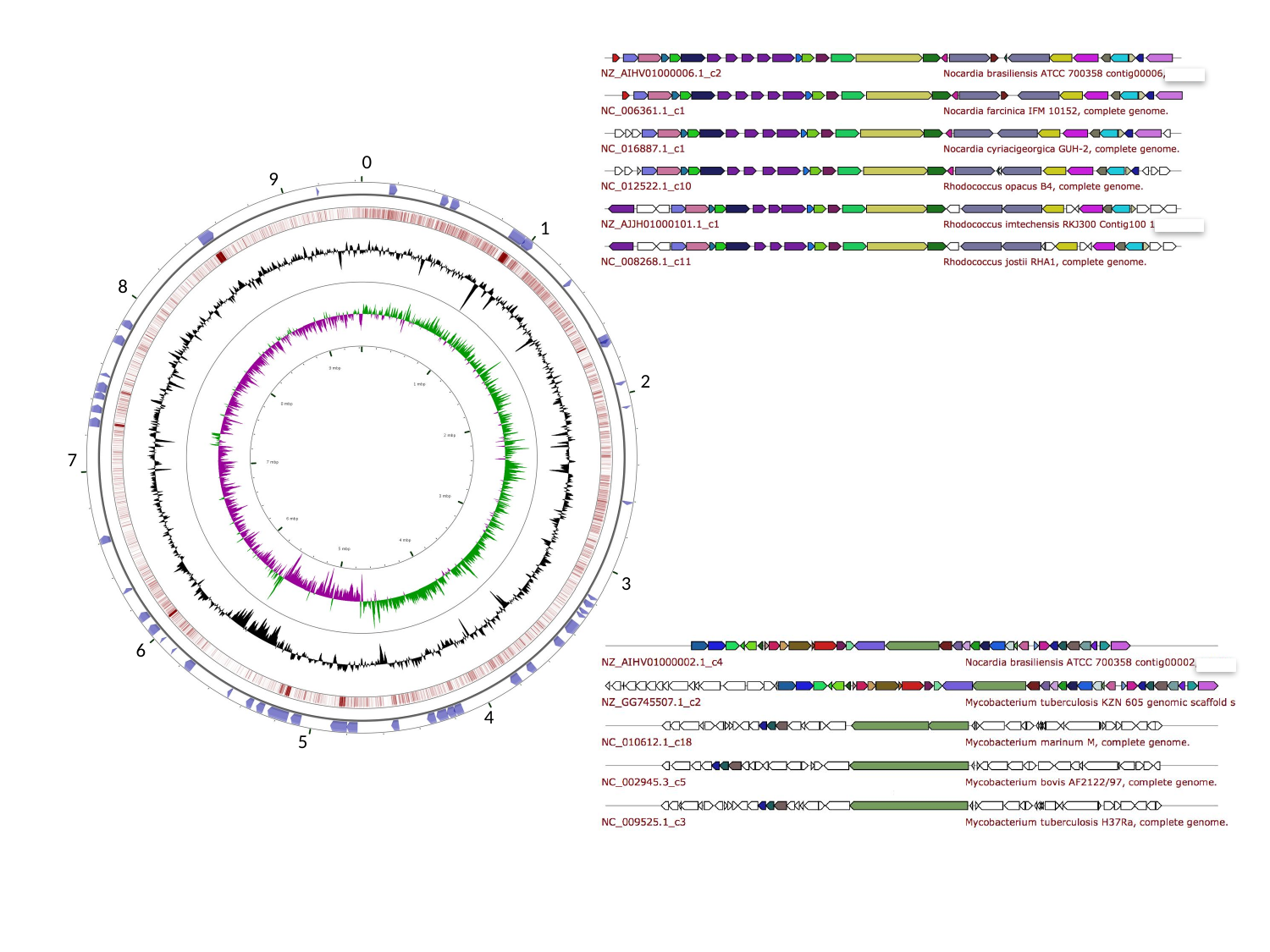

0
9
1
8
2
7
3
6
4
5

Supplement: Figure S1 — Genomic location of the 47 gene clusters found in Nocardia brasiliensis HUJEG-1 using Antibiotics and Secondary Metabolites Analysis Shell (antismash) software [http://antismash.secondarymetabolites.org]. Besides the map we show a comparative analysis of cluster 1, which is highly conserved among the Nocardiaceae, located between nucleotides 1682661 – 1725128 nt, and cluster 38, which is quite specific of N. brasiliensis and is located between nucleotides 6591347–6636992 (PPTX) [file pone.0065425.s001.pptx]
