# Supplementary material for: Complete Genome Sequence Analysis of Nocardia brasiliensis HUJEG-1 Reveals a Saprobic Lifestyle and the Genes Needed for Human Pathogenesis
Source: PLoS One. 2013 Jun 3;8(6):e65425. doi: 10.1371/journal.pone.0065425 (PMC3670865; doi:10.1371/journal.pone.0065425)
Supplement: Table S1 — Presence of ortholog genes of putative virulence factors of N. brasiliensis in other microorganisms. (DOCX) [file pone.0065425.s002.docx]

| **NAME** | **LOCUS TAG** | **PROTEIN PRODUCT** | **PERCENT COVERAGE/% IDENTITY** | |  |
| --- | --- | --- | --- | --- | --- |
|  |  |  | **NF** | **NC** | **Closest matching organism** |
| Catalase | O3I_001640 | YP_006805265.1 | - | 98/87 | *Mycobacterium abscessus subsp. Bolleti* 98/85 |
| Catalase/hydroperoxidase HPI(I) | O3I_014530 | YP_006807841.1 | 100/73 | - | *Mycobacterium haemophilum* 99/77 |
| Catalase/hydroperoxidase HPI(I) | O3I_018595 | YP_006808652.1 | - | - | *Gordonia sputi*  99/79 |
| Catalase | O3I_021945 | YP_006809318.1 | - | - | *Rhodococcus opacus* 97/71 |
| Catalase | O3I_032795 | YP_006811482.1 | - | - | *Rhodococcus opacus* 96/55 |
|  |  |  |  |  |  |
| Superoxide dismutase | O3I_000385 | YP_006805014.1 | 99/97 | 100/96 | *Mycobacterium thermoresistibile* 100/95 |
| Superoxide dismutase | O3I_039690 | YP_006812859.1 | 100/74 | 100/68 | *Rhodococcus equi*  99/66 |
|  |  |  |  |  |  |
| Phospholipase C | O3I_010265 | YP_006806988.1 | - | - | *Amycolaptosis mediterranei*  99/54 |
| Phospholipase C | O3I_012930 | YP_006807521.1 | - | - | *Gordonia effusa* 99/75 |
| Phospholipase C | O3I_019520 | YP_006808837.1 | - | - | *Amycolaptosis azurea* 98/61 |
| Phospholipase C | O3I_025065 | YP_006809938.1 | - | - | *Granulicella mallensis*  98/61 |
| Lysophospholipase | O3I_025675 | YP_006810060.1 | 95/65 | - | *Mycobacterium gilvum*  98/67 |
| Non-hemolytic phospholipase C | O3I_029305 | YP_006810784.1 | - | - | *Mycobacterium massilliense*  90/67 |
| Phospholipase D | O3I_032765 | YP_006811476.1 | - | - | *Thiomonas sp.*  77/33 |
| Phospholipase | O3I_033135 | YP_006811550.1 | - | - | *Streptomyces roseosporus* 90/78 |
| Putative lysophospholipase | O3I_035450 | YP_006812011.1 | 97/67 | 94/69 | *Rhodococcus jostii* 90/45 |
|  |  |  |  |  |  |
| Hemolysin-type calcium-binding protein | O3I_012605 | YP_006807456.1 | - | - | *Actinomyces sp*  100/43 |
| Cytotoxin/hemolysin | O3I_013705 | YP_006807676.1 | 100/83 | - | *Rhodococcus pyridinivorans* 100/77 |
| Membrane channel protein, hemolysin III | O3I_036360 | YP_006812193.1 | 99/81 | 92/85 | *Rhodococcus erythropolis* 91/71 |
| Hemolysin | O3I_037730 | YP_006812467.1 | - | - | *Renibacterium salmoninarum*  80/45 |
|  |  |  |  |  |  |
| Putative membrane-bound rhomboid protease | O3I_000100 | YP_006804957.1 | 89/63 | 98/79 | *Rhodococus wratislaviensis* 98/59 |
| Putative Trypsin-like serine protease | O3I_001810 | YP_006805299.1 | 97/89 | 100/88 | *Rhodococcus equi* 99/75 |
| Clp protease | O3I_002340 | YP_006805405.1 | 97/98 | 93/99 | *Rhodococcus piridinovorans*  96/96 |
| Clp protease | O3I_002345 | YP_006805406.1 | 987/87 | 96/88 | *Rhodococcus piridinovorans* 96/68 |
| Protease II, ptrB | O3I_003115 | YP_006805560.1 | 97/85 | 99/85 | *Rhodococcus erythropolis*  97/78 |
| Putative protease | O3I_003200 | YP_006805577.1 | 83/53 | 85/61 | *Rhodococcus opacus*  91/41 |
| Putative Membrane-anchored subtilisin-like serine protease (modular protein) | O3I_004700 | YP_006805877.1 | 97/71 | 99/72 | *Gordonia soli*  95/56 |
| Protease | O3I_004845 | YP_006805906.1 | 99/67 | 98/69 | *Rhodococcus opacus*  97/52 |
| ATP-dependent Clp protease adaptor protein ClpS | O3I_006455 | YP_006806228.1 | 100/85 | 84/95 | *Rhodococcus erythropolis*  85/80 |
| ATP-dependent Clp protease proteolytic subunit | O3I_008810 | YP_006806699.1 | 98/94 | 92/83 | *Rhodococcus erythropolis*  99/88 |
| ATP-dependent Clp protease proteolytic subunit | O3I_008815 | YP_006806700.1 | 98/95 | 100/94 | *Rhodococcus opacus*  100/90 |
| ATP-dependent protease ATP-binding subunit ClpX | O3I_008820 | YP_006806701.1 | 100/96 | 96/97 | *Rhodococcus jostii*  100/95 |
| Membrane protease subunit stomatin/prohibitin-like protein | O3I_011090 | YP_006807153.1 | - | - | *Amycolatopsis mediterranei*  85/76 |
| CAAX amino terminal protease family protein | O3I_013035 | YP_006807542.1 | - | - | *Micavibrio aeruginosavorus*  93/37 |
| ATP-dependent Clp protease | O3I_013280 | YP_006807591.1 | - | - | *Bifidobacterium angulatum*  92/49 |
| Protease | O3I_016845 | YP_006808304.1 | 97/73 | - | *Amycolicicoccus subflavus* 93/67 |
| Serine protease | O3I_019690 | YP_006808871.1 | - | 99/87 | *Saccharomonospora glauca*  97/74 |
| Clp protease | O3I_023275 | YP_006809584.1 | 62/93 | 62/95 | *Saccharothrix espanoensis*  98/67 |
| Trypsin-like serine protease | O3I_027530 | YP_006810429.1 | - | - | *Saccharopolyspora erythraea*  86/41 |
| Metalloprotease | O3I_028500 | YP_006810623.1 | 100/77 | 99/75 | *Rhodococcus opacus*  100/70 |
| Protease | O3I_030410 | YP_006811005.1 | 97/92 | 99/88 | *Rhodococcus equi*  97/79 |
| Zinc metalloprotease | O3I_030935 | YP_006811110.1 | 99/73 | 100/86 | *Rhodococcus equi* 100/62 |
| ATP-dependent Clp protease proteolytic subunit | O3I_033990 | YP_006811721.1 | - | - | *Rhodococcus erythropolis* 97/62 |
| ATP-dependent Clp protease proteolytic subunit ClpP | O3I_033995 | YP_006811722.1 | - | - | *Rhodococcus opacus* 97/79 |
| Protease inhibitor protein | O3I_034615 | YP_006811846.1 | - | - | *Streptomyces viridochromogenes* 100/46 |
| Putative protease | O3I_035790 | YP_006812079.1 | 100/76 | 100/79 | *Rhodococcus jostii* 94/63 |
| Putative protease | O3I_037505 | YP_006812422.1 | 95/75 | 85/77 | *Rhodoccus opacus* 100/57 |
| Putative Clp protease subunit Probable ATP-dependent Clp protease ATP-binding subunit | O3I_038700 | YP_006812661.1 | 98/68 | 100/70 | *Rhodococcus opacus* 100/52 |
| Putative serine protease | O3I_039125 | YP_006812746.1 | 98/68 | 96/69 | *Mycobacterium xenopi* 86/42 |
| Putative Clp protease subunit | O3I_039575 | YP_006812836.1 | 98/68 | 100/73 | *Rhodococcus equi* 100/66 |
| Protease | O3I_040345 | YP_006812988.1 | 99/80 | 95/82 | *Rhodococcus erythropolis* 99/57 |
| Putative secreted protease | O3I_040350 | YP_006812989.1 | 100/78 | 98/81 | *Rhodococcus piridinovorans* 99/51 |
| ATP-dependent protease La | O3I_041840 | YP_006813287.1 | 100/90 | 100/89 | *Gordonia amicalis* 98/73 |
